# Characterization of Carbon Dust on the Anode Surface in the Hall–Héroult Process

**DOI:** 10.3390/ma19132774

**Published:** 2026-06-30

**Authors:** Stanisław Pietrzyk

**Affiliations:** Department of Physical Chemistry and Metallurgy of Non-Ferrous Metals, Faculty of Non-Ferrous Metals, AGH University of Krakow, Al. Adama Mickiewicza 30, 30-059 Kraków, Poland; pietstan@agh.edu.pl

**Keywords:** aluminium electrolysis, Hall-Héroult process, carbon dust, anode effect, electrophoresis, anode spikes, SEM-EDS, XRD, Raman spectroscopy

## Abstract

This study provides a comprehensive characterization of carbon dust adhesion on the anode surface induced by the anode effect (AE) in the Hall–Héroult process. The primary objective was to verify the hypothesis of electrophoretic carbon particle transport and its subsequent stabilization on the electrode substrate. Unlike previous studies conducted in horizontal configurations where gravitational sedimentation could interfere with observations, this research employs a unique vertical electrode setup to provide direct physical evidence of purely electrophoretic transport. Authentic industrial carbon dust was used as a tracer material, its presence on the high-purity graphite surface being definitively confirmed through the detection of trace markers (Mg, Ca) via SEM-EDS. The multiscale structural analysis revealed that spike initiation occurs through a dynamic arc-induced nucleation mechanism. Morphological observations suggest that micro-arc discharges during the AE provide the extreme localized energy for direct carbon-to-carbon “welding,” creating a conductive, porous scaffold on the vertical anode wall. XRD analysis identified crystalline cryolite (Na_3_AlF_6_) and chiolite (Na_5_Al_3_F_14_) within this structure. It was demonstrated that these fluoride phases represent the solidified product of molten, acidic electrolyte infiltration into the carbonaceous matrix via capillary action, rather than acting as binders that crystallize during the process. Raman spectroscopy confirmed the disordered, amorphous nature of the captured dust (high D-band intensity), distinguishing it from the highly ordered graphite substrate. Confocal microscopy visualized the topographical evolution from isolated clusters to interconnected three-dimensional “islands” as a function of AE duration. The results demonstrate that the anode effect serves as a critical flashpoint where synergistic electrophoretic forces and localized thermal anomalies initiate the growth of stable, conductive carbon–matrix composite spikes, providing new insights for mitigating current efficiency losses in industrial smelters.

## 1. Introduction

### 1.1. Background of the Hall–Héroult Process and Anode Dusting

The Hall–Héroult process, discovered independently in 1886, remains the only industrial method for the primary production of aluminum [1]. This electrochemical process involves the electrolytic reduction of alumina (Al_2_O_3_) dissolved in a molten bath consisting mainly of cryolite (Na_3_AlF_6_) at temperatures typically ranging between 950 °C and 970 °C [2]. The reduction takes place in specifically designed electrolytic cells, where carbon anodes are consumed to react with the released oxygen, primarily producing carbon dioxide gas. While the technology has undergone significant engineering refinements over the last century, the fundamental principles of molten salt electrolysis remain unchanged [3]. The process is characterized by high energy intensity and a delicate balance between electrochemical kinetics, magnetohydrodynamics, and thermal stability. Maintaining a stable interpolar distance and optimal electrolyte chemistry is critical for maximizing current efficiency and minimizing environmental emissions [4,5].

**Figure 1:**
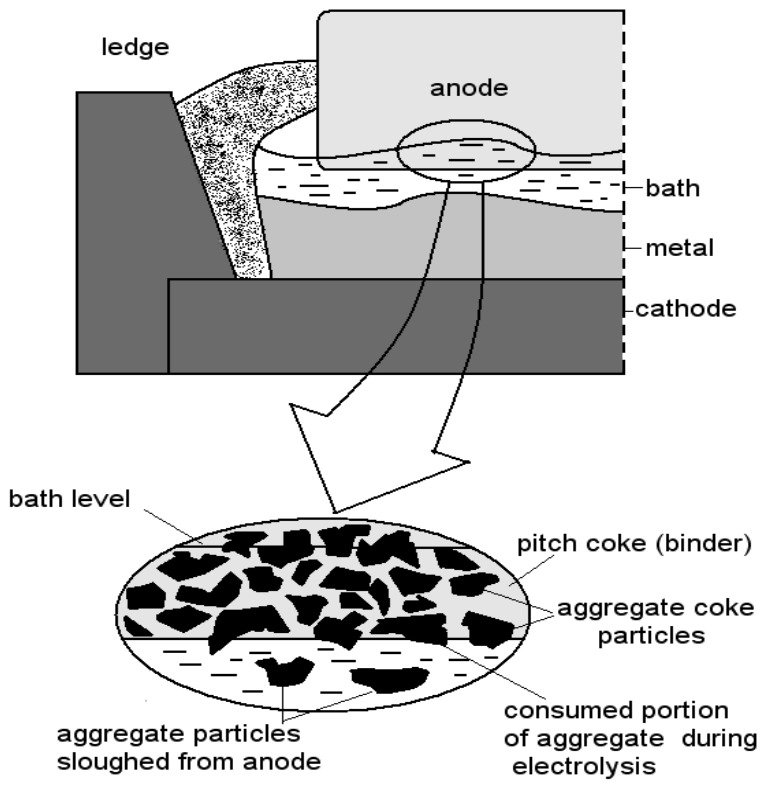
Schematic of the anode consumption mechanism illustrating the phenomenon of anode dusting, where preferential oxidation of the pitch coke binder leads to the mechanical detachment and sloughing of aggregate carbon particles into the cryolite bath.

Optimizing the Hall–Héroult process remains a priority for global energy reduction in the aluminum industry [1,3]. One of the most persistent challenges to cell stability is the phenomenon of “anode dusting,” characterized by the release of carbon particles from the anode into the molten cryolite electrolyte. This carbon dust originates primarily from the selective oxidation of the pitch binder and the subsequent mechanical disintegration of the coke aggregate (Figure 1). Once suspended in the bath, this dust forms a “carbon foam” layer, altering the electrolyte’s physicochemical properties and significantly increasing electrical resistance [5,6].

**Figure 2:**
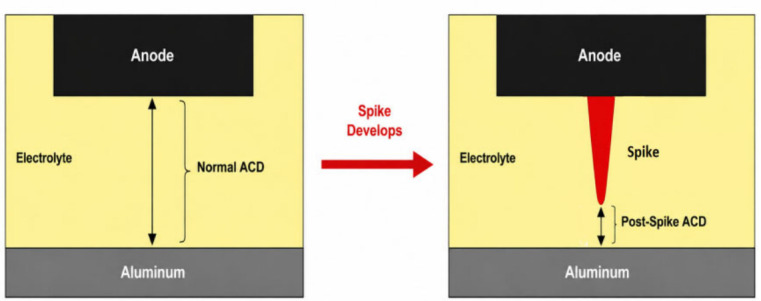
Spike formation on anode in Hall–Héroult cells.

The presence of carbon dust is the direct precursor to the formation of anode spikes—stable, conductive carbonaceous growths that bridge the interpolar gap (ACD) (Figure 2). These spikes are a major operational threat, leading to localized current maldistribution, increased heat generation, and frequent metallic short circuits. For industrial smelters, this results in a drastic reduction in current efficiency and increased greenhouse gas emissions. Despite decades of research, the precise physical mechanism that transforms loose, suspended dust into a permanent, “welded” anode growth remains a subject of intense scientific debate [7,8].

### 1.2. Research Hypothesis and Objectives

The primary research hypothesis of this study assumes that the rapid increase in electric field intensity during the anode effect (AE) induces an intensive electrophoretic transport of negatively charged carbon dust particles toward the anode surface. This process leads to their permanent adhesion and subsequent stabilization via arc-induced nucleation.

To verify this, a novel experimental setup was designed using vertical graphite electrodes in a parallel arrangement (detailed in Section 3.2 and demonstrated via schematic and photographic views in Figure 4). Unlike previous studies conducted in horizontal configurations, where gravitational sedimentation could interfere with observations, this research employs a unique vertical electrode setup to provide the first direct physical evidence of purely electrophoretic carbon dust transport. This configuration is intended to eliminate gravitational settling as a factor, ensuring that any carbon buildup on the vertical wall is a direct result of electric field forces [9].

The novelty of this study is further enhanced by several key methodological and theoretical advancements:

- Industrial Tracer Methodology: Using authentic industrial skimmings as a tracer, we utilized Mg and Ca chemical markers to definitively distinguish redeposited dust from primary anode material, following a structural and chemical tracing approach [10].
- Arc-Induced Nucleation Model: We propose a shift from simple adhesion theories to a dynamic model where micro-arc discharges during the AE act as the “welding” energy. These high-energy plasma events facilitate the fusion of carbon dust particles into a permanent, conductive scaffold [9,11].
- Composite Scaffold Infiltration: The model demonstrates that the initiated spike is a carbon–electrolyte composite, where the porous carbon scaffold is subsequently infiltrated by an acidic fluoride melt.

The main objective of the research is to provide a multiscale characterization of these secondary carbon deposits. The experimental verification involves an integrated analytical approach, including Scanning Electron Microscopy with Energy Dispersive Spectroscopy (SEM-EDS) for morphological and chemical mapping, X-ray Diffraction (XRD) for phase identification of the electrolyte infiltrant, and Raman spectroscopy combined with confocal microscopy to evaluate the structural ordering and spatial distribution of the captured industrial dust [12,13].

## 2. Literature Review

### 2.1. Evolution of Spike Formation Theories

Scientific discourse regarding the initiation of anode spikes has evolved through several distinct paradigms over the past decades. Early research was dominated by the “Carbide Stalactite” theory [14,15], which suggested that spikes were primarily products of chemical synthesis—specifically the formation of aluminum carbide at the anode interface. This was subsequently challenged by the “Mechanical Entrapment” model [8,16], which viewed spikes as results of physical debris capture and gravitational settling.

More recently, the “Vicious Circle” theory [7] highlighted the thermal feedback loops: a localized deposit reduces the interpolar gap, increasing current density and heat, which in turn accelerates further carbon capture. However, most of these studies were conducted in horizontal cell configurations, where gravitational sedimentation often masked the subtle electrokinetic forces, leaving the initial “anchoring” mechanism of the dust particles unresolved [9].

The transport of suspended carbon dust in the electrolyte is primarily governed by electrokinetic phenomena. This negative surface charge originates from the preferential adsorption of complex fluoride anions (such as AlF_6_^3−^) onto the carbon surface, forming an electrical double layer where the net negative charge of the particle is macroscopically balanced by a surrounding diffuse cloud of sodium counter-ions (Na^+^) [9]. Carbon particles in molten fluoride salts acquire a surface charge (zeta potential), the magnitude and sign of which depend on the bath chemistry and the cryolite molar ratio (CR). Polyakov [9] demonstrated that under industrial conditions, carbon particles exhibit significant electrophoretic mobility. When the electric field intensity surges during an anode effect (AE), these negatively charged particles are subjected to intense horizontal forces. Unlike gravitational settling, this electrophoretic migration is capable of transporting dust across the interpolar gap to the vertical faces of the anode, establishing the primary contact required for spike initiation.

The transition from a loose particle to a “welded” deposit is facilitated by the extreme conditions of the anode effect. The formation of a continuous, insulating gas film leads to the dielectric breakdown of the interface, resulting in micro-arc discharges and localized plasma formation [9,11].

These high-energy plasma events provide a massive localized thermal density, which we propose acts as the “welding energy”. This process, termed arc-induced nucleation, allows the carbon particles to fuse with the anode substrate and with each other, forming a conductive scaffold. Furthermore, the localized depletion of alumina during the AE leads to the acidification of the melt. As established by Thonstad et al. and Robelin et al. [2,17], this acidic environment promotes the stability of the chiolite phase, which subsequently infiltrates the porous carbon scaffold, providing the final structural robustness to the composite spike.

### 2.2. Structural Characterization of Carbonaceous Matter

The evaluation of redeposited carbon requires a distinction between the highly ordered graphite of the anode and the disordered nature of the industrial dust. Raman spectroscopy serves as a critical tool in this differentiation. According to Sadezky et al. and Ferrari and Robertson [12,18], the relative intensities of the D-band (disorder-induced) and G-band (graphitic) provide a structural fingerprint of the carbon’s origin. Similarly, XRD analysis allows for the identification of the electrolyte infiltrant, where the presence of chiolite serves as a marker for the acidic conditions prevalent during the nucleation process [10,13].

## 3. Experimental Methodology

### 3.1. Materials and Industrial Dust Tracers

High-purity isostatic graphite (provided by SGL CARBON POLSKA S.A., Racibórz, Poland) was selected as the model material for both the anode and cathode to ensure maximum analytical precision and to eliminate the complexities associated with porous industrial substrates. Unlike industrial prebaked anodes, which exhibit high primary roughness and heterogeneous binder–aggregate interfaces, the dense and homogeneous structure of isostatic graphite provides a characteristic smooth surface. This surface quality is a critical component of the experimental strategy, as it facilitates the unambiguous identification of redeposited carbon dust via SEM-EDS techniques by eliminating topographical interference and background noise from the electrode’s own microstructure.

**Figure 3:**
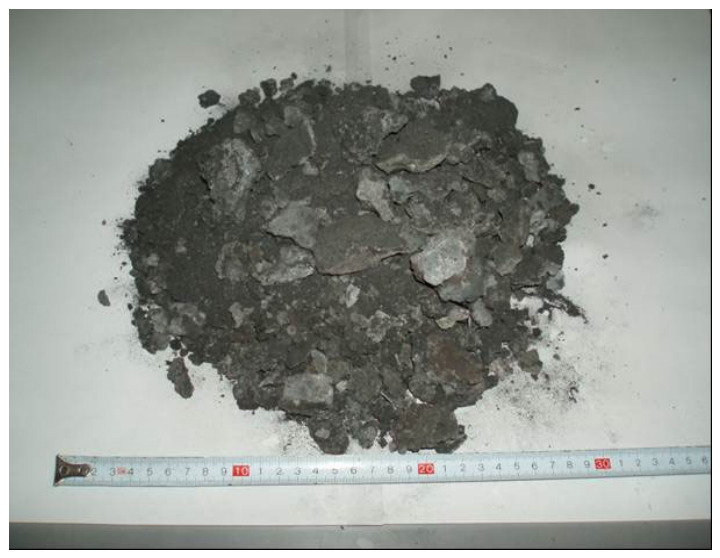
Raw industrial carbon dust skimmed from an operational Hall–Héroult cell, utilized as a natural tracer for the experimental investigation of electrophoretic transport.

To faithfully replicate the operational environment of an industrial smelter, authentic carbon dust was collected from the carbon foam layer (skimmings) of a high-amperage operational aluminum electrolyzer. This raw material (Figure 3) was sampled directly from the electrolyte surface to serve as a natural tracer in the experiment. The physical form of the dust, consisting of fragmented coke particles of varying sizes, provides a realistic representation of the carbonaceous matter suspended in industrial baths. The typical physicochemical composition of the collected industrial skimmings consisted of 55–65 wt.% cryolite (Na_3_AlF_6_), 15–20 wt.% carbonaceous matter (C), 6–9 wt.% aluminum fluoride (AlF_3_), 4–8 wt.% alumina (Al_2_O_3_), 1–2 wt.% magnesium fluoride (MgF_2_) and 2–4 wt.% calcium fluoride (CaF_2_). Because the source tracer contains only 2–4 wt.% of CaF_2_, the baseline weight fraction of elemental Calcium within the bulk industrial foam is chemically restricted to approximately 1.0–2.0 wt.%, with Magnesium existing in even lower trace quantities.

As demonstrated by Bugnion and Fischer et al. and Ishak et al. [10,13], industrial skimmings possess a unique “chemical signature” because the coke particles become deeply embedded and impregnated with electrolyte additives, such as Mg and Ca, during their prolonged residence in the molten bath. The use of such pre-characterized industrial dust allows for a definitive differentiation between graphite electrode degradation products and the exogenous material (carbon dust). Consequently, any detection of Mg and Ca markers on the high-purity graphite surface following the anode effect provides conclusive evidence that the observed deposits originate from the suspended industrial dust captured during the arc-induced nucleation process.

### 3.2. Experimental Setup

**Figure 4:**
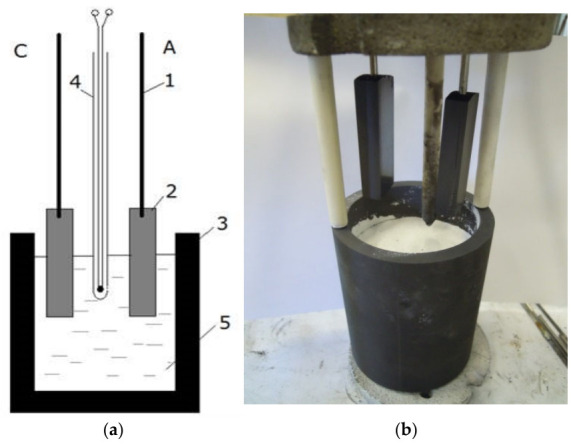
Experimental electrolysis system: (**a**) Schematic diagram of the experimental cell: 1—stainless steel rods, 2—graphite electrodes (A: anode, C: cathode), 3—graphite crucible, 4—shielded thermocouple, 5—molten electrolyte; (**b**) Photographic documentation of the assembled electrolytic cell before immersion.

The experimental setup was engineered with a unique vertical orientation of the graphite electrodes, a configuration specifically designed to isolate electrokinetic phenomena from gravitational influences (Figure 4). The implementation of this parallel vertical arrangement, where the anode and cathode are immersed vertically into the electrolyte, is a critical methodological choice. In traditional horizontal laboratory cells, carbon dust particles tend to settle on the electrode surfaces due to simple sedimentation, making it impossible to distinguish between mechanical settling and active electrokinetic transport.

By positioning the electrodes vertically, the gravitational vector acts parallel to the working faces rather than perpendicular to them. As established in the fundamental studies by Polyakov at al. [9], such a configuration is essential to eliminate the “sedimentation bias” that frequently masks subtle electrokinetic forces in molten salts. Consequently, any carbon accumulation detected on the vertical wall of the anode serves as a direct and unambiguous physical proof of horizontal electrophoretic transport driven exclusively by the electric field intensity.

The assembly allowed for the carbon dust to migrate horizontally across the 4.5 cm interpolar gap, providing high clarity in observing the influence of electric field forces during the forced induction of the anode effect. The entire cell was housed in a precision high-temperature furnace, ensuring a stable thermal environment for the subsequent arc-induced nucleation and structural stabilization of the redeposited dust particles.

### 3.3. Electrolyte Composition and Cell Parameters

The investigations were conducted using a strictly controlled synthetic electrolyte prepared from high-purity analytical grade reagents (purity > 99%, Sigma-Aldrich, Burlington, MA, USA). The bath consisted of a mass composition of 87.3 wt.% Na_3_AlF_6_ and 12.7 wt.% AlF_3_, resulting in a cryolite molar ratio (CR) of 2.2. To ensure the rapid and stable induction of the anode effect, the electrolyte was prepared without the addition of alumina (Al_2_O_3_). As established by Thonstad et al. [2,19], the absence of alumina combined with a lower cryolite ratio (acidic bath) dramatically increases the susceptibility of the cell to voltage anomalies and the formation of a continuous gas film. These parameters are necessary conditions for studying the electrokinetic migration of carbonaceous matter under extreme electric field intensities.

A critical component of the experimental matrix was the addition of 5 wt. % of authentic industrial carbon dust (skimmings) directly into the molten bath. This concentration was selected based on the findings of Bugnion & Fischer and Pietrzyk & Thonstad [5,10] to provide a sufficient reservoir of particles for electrophoretic capture while maintaining the fluid properties and density of the acidic electrolyte.

The process temperature was strictly maintained at 975 °C, ensuring a stable “superheat” relative to the liquidus temperature of the melt [1,2]. The graphite electrodes with working dimensions of 20 mm in length and 9 mm in width were immersed to a depth of 30 mm, providing a well-defined surface area for the subsequent arc-induced nucleation. Current was supplied via stainless steel rods, and thermal stability was continuously monitored using a shielded thermocouple positioned centrally within the electrolyte. This specific chemical and thermal environment was designed to maximize the intensity of the electric field forces acting on the suspended carbon dust particles, while simultaneously maintaining physicochemical properties closely resembling those of industrial electrolytes during localized alumina depletion.

### 3.4. Procedure for AE Induction and Sample Preservation

Electrolysis was conducted under galvanostatic conditions at an identical anodic and cathodic current density of 1.04 A/cm^2^ (constant current of 10 A DC). To evaluate the kinetics of the process and the structural evolution of the deposits, two distinct AE durations were applied: 60 min and 120 min.

**Figure 5:**
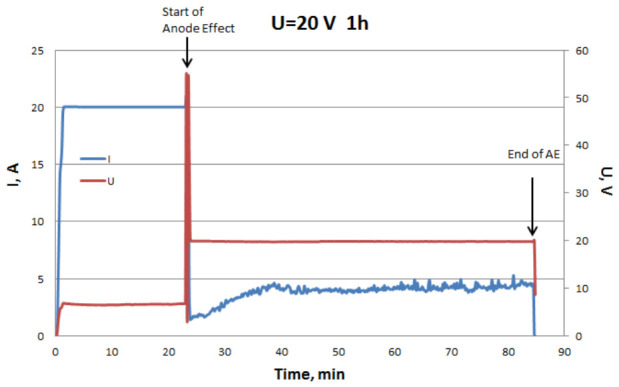
Temporal changes in voltage (red line) and current (blue line) during the 60 min anode effect experiment (Experiment No. 1).

**Figure 6:**
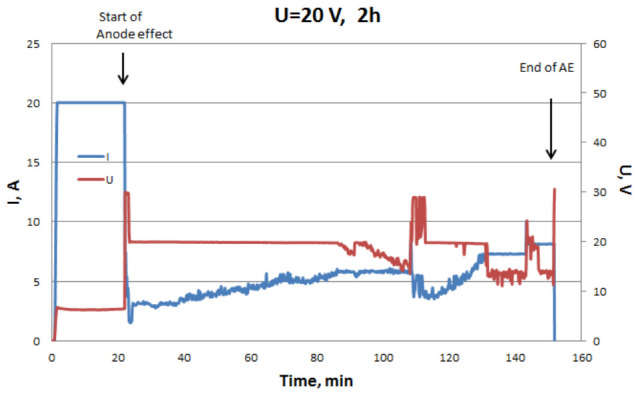
Temporal changes in voltage (red line) and current (blue line) during the 120 min anode effect experiment (Experiment No. 2).

Figure 5 and Figure 6 illustrate the temporal changes in voltage (red line) and current (blue line) for these two experimental trials. As shown in the plots, the initiation of the AE is marked by a sudden, sharp voltage surge and a simultaneous collapse in current. These electrical characteristics and the resulting stability of the process under controlled potential are consistent with the observations of voltage and current oscillations during the anode effect in fluoride melts [20]. Immediately following this onset, the imposed voltage was rapidly lowered and stabilized. The experimental equipment was specifically capable of holding the anode effect active at a reduced potential of 20 V for the entire designated duration (Experiment No. 1 for 60 min or Experiment No. 2 for 120 min).

Upon completion of the designated time, a specialized preservation protocol was implemented:

- The anode was lifted into the furnace’s cool zone while the 20 V potential remained active.
- As emphasized by Ishak et al. [13], this is essential to counteract the high surface tension of the cryolite melt, ensuring the newly formed composite layers and infiltrated electrolyte remain in their representative state.
- The entire cell was subsequently cooled to ambient temperature under a strictly controlled argon atmosphere.

According to the analytical protocols established by Sadezky et al. [12], this rapid quenching and protection from airburn oxidation are vital for maintaining the structural integrity of the carbon surface. This ensured that subsequent SEM-EDS, XRD, and Raman investigations provided a reliable representation of the arc-induced nucleation and the true structural disorder of the redeposited industrial dust.

### 3.5. SEM-EDS Characterization

Microstructural evaluation and chemical mapping of the pristine graphite and the post-test redeposited carbon layers were performed using a scanning electron microscope (SEM, JEOL JSM-6610LV, Akishima, Japan) equipped with an Oxford Instruments energy-dispersive X-ray spectrometer (EDS). To resolve both the fine carbon dust boundaries and the heavier electrolyte phase constituents, imaging was conducted at an accelerating voltage of 15–20 kV under high-vacuum conditions with a working distance maintained at 10 mm. Quantitative point analyses (pt1–pt8) and elemental spatial distribution maps for Carbon (C), Fluorine (F), Sodium (Na), Aluminum (Al), Magnesium (Mg), and Calcium (Ca) were acquired using an acquisition live time of 60 s per spectrum to ensure a high signal-to-noise ratio for the trace industrial markers.

### 3.6. X-Ray Diffraction (XRD) Analysis

Phase identification of the raw industrial tracer and the multi-phase material scraped from the vertical anode surfaces was carried out via X-ray diffraction (XRD). The measurements were executed on a high-resolution diffractometer utilizing monochromatic (Cu-Kα radiation λ = 1.5418 Å) operating at 40 kV and 40 mA. Data collection was performed at room temperature over a 2θ scanning range from 10° to 80° with a step size of 0.02° and a counting time of 2 s per step. Phase identification and matching of the amorphous coke background, crystalline cryolite (Na_3_AlF_6_), and chiolite (Na_5_Al_3_F_14_) reflections were achieved using standard crystallographic databases.

### 3.7. Raman Spectroscopy Analysis

The structural ordering and allotropic fingerprinting of the carbonaceous matter were evaluated via Raman spectroscopy using a high-resolution achromatic reflective Raman microscope (LabRAM HR, Horiba Jobin Yvon, Lille, France) equipped with an Olympus BX-41 optical microscope (Olympus Corp., Tokyo, Japan). Raman scattering was excited using a solid-state diode laser with a wavelength of 532 nm (green light), which provides an optimal balance between carbon scattering efficiency and the minimization of background fluorescence. The laser beam was focused through a 50× long-working-distance metallurgical objective, yielding a sub-micron lateral spot size (approximately 1.0 µm). To prevent localized laser-induced thermal graphitization or photo-oxidation of the disordered amorphous carbon phases, the laser power at the sample surface was strictly attenuated to below 5 mW.

The scattered light was dispersed using a high-density holographic grating with 1800 lines/mm and detected with a thermoelectrically cooled charge-coupled device (CCD) detector. Spectra were recorded in the wavenumber range of 100–3500 cm^−1^ to capture both the low-frequency mineral lattice modes and the carbonaceous first- and second-order bands (D, G, and 2D). Each spectral acquisition was performed with an exposure time of 10 s and averaged over 3 accumulations to maximize the signal-to-noise ratio. Multi-point polynomial baseline subtraction and curve fitting using Gaussian–Lorentzian functions were consistently applied to isolate genuine vibrational frequencies from residual amorphous luminescence.

### 3.8. Laser Confocal Microscopy Profiling

Topographical imaging and three-dimensional spatial tracking of the integrated mineral phases within the carbonaceous matrix were complementarily visualized using a high-resolution laser confocal microscope, LEXT OLS4100 Olympus, Hachiōji, Japan), operating in reflected light. The system operated using a dual-channel setup: a polarized light channel for optical contrast and mineral phase differentiation, and a laser scanning channel utilizing a 405 nm violet laser diode for high-precision surface topography profiling.

The objective lens configuration (20× and 50× magnification) combined with a motorized Z-axis stage allowed for a high vertical resolution (Z-axis step size of 0.1 µm). This enabled the accurate extraction of surface roughness profiles and the documentation of the architectural transition from isolated carbon clusters to massive, interconnected three-dimensional composite structures. The acquired data were processed using dedicated surface analysis software to generate high-contrast multi-color topographical maps of the redeposited layers and infiltrated electrolyte channels.

## 4. Results and Discussion

### 4.1. Macroscopic Observations of the Anode and Cathode

**Figure 7:**
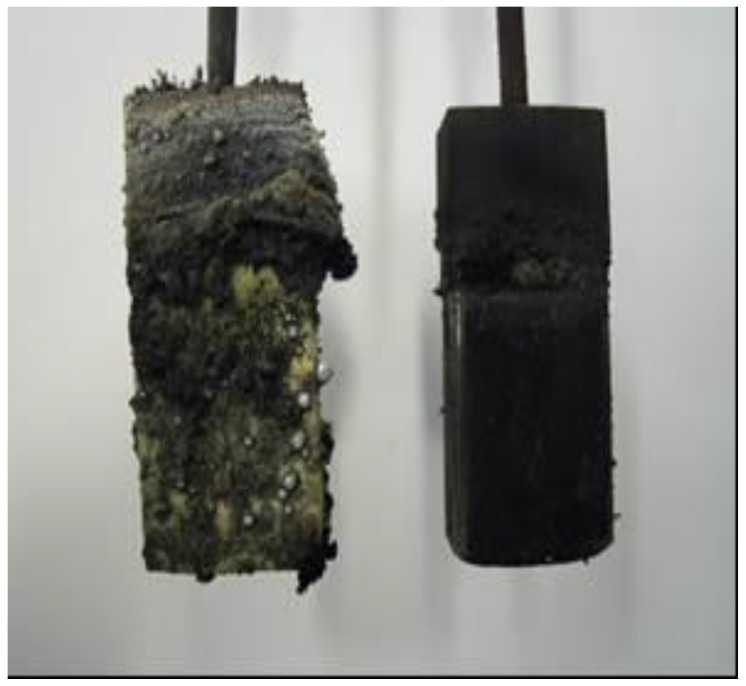
Macroscopic documentation of the electrodes after the electrolysis test: (**left**) cathode surface with metallic aluminum droplets; (**right**) anode surface with redeposited carbon dust agglomerates on the vertical face.

Visual inspection of the electrodes following the electrolysis tests revealed distinct and contrasting morphological changes on both surfaces, providing immediate qualitative evidence of the differing electrochemical processes (Figure 7).

On the cathode (Figure 7, left), the surface was characterized by the presence of numerous metallic aluminum droplets of varying sizes. A distinct, yellowish-gray crust was observed at the interface, which serves as a macroscopic indicator suggestive of aluminum carbide (Al_4_C_3_) formation, consistent with typical cathode–melt interactions documented in the literature [2]. While a definitive instrumental phase validation (such as XRD or SEM-EDS) of this cathode layer was outside the scope of this study and not performed, the upper part of the cathode remained relatively clean, with no signs of exogenous carbon.

On the anode (Figure 7, right), the surface exhibited a radically different morphology. The vertical working face was almost entirely covered by irregular, dark, and highly robust deposits. The appearance of these carbonaceous coatings specifically on the vertical wall—rather than settling at the bottom of the crucible—provides the most compelling macroscopic support for the electrophoretic transport hypothesis. Under normal conditions, gravitational forces would prevent suspended dust from adhering to a vertical surface. The fact that the dust bypassed horizontal surfaces and anchored itself to the vertical graphite face proves that the intense electric field during the anode effect (AE) provided the necessary horizontal force to overcome gravitational sedimentation [9].

Furthermore, the deposits on the anode appear as a dense, “crusty” composite rather than loose particles. This macroscopic robustness suggests that the carbon dust was not merely trapped but was stabilized through arc-induced nucleation. The preservation of these fragile yet stable structures was successfully achieved through the 20 V maintenance and argon cooling procedure, which “froze” the infiltrated electrolyte within the carbon scaffold, allowing for further high-resolution analysis of the captured industrial dust particles.

### 4.2. SEM-EDS Analysis of Carbon Deposits and Markers

**Figure 8:**
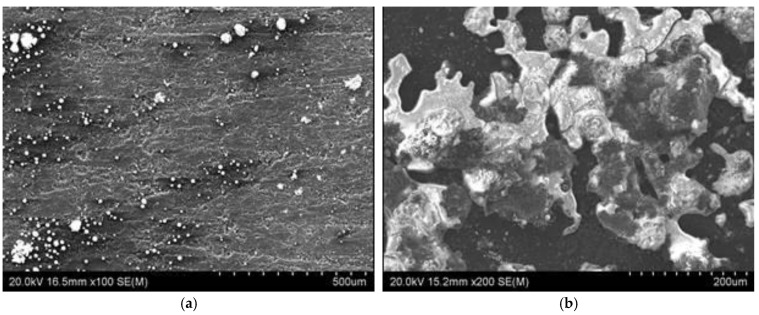
SEM micrographs of the graphite anode surface: (**a**) non-immersed section showing the smooth, primary isostatic graphite structure; (**b**) working anode surface illustrating the early stage of carbon dust adhesion and the “polishing” effect induced by the micro-arc discharges during the AE.

High-resolution SEM imaging provided definitive evidence of the morphological and chemical transformation of the anode surface during the AE. A comparative analysis reveals a radical difference between the non-immersed and working sections of the electrode (Figure 8). While the non-immersed section represents the smooth, baseline state of the isostatic graphite (Figure 8a), the working section exhibits a complex, multi-layered topography that documents the progression of carbon dust accumulation (Figure 8b).

### 4.3. Morphological Evolution and Nucleation

**Figure 9:**
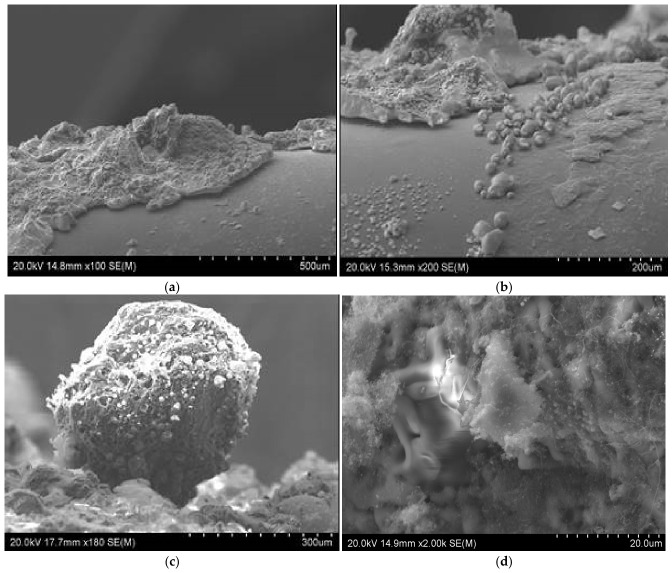
High-magnification SEM imaging documenting the progression of spike initiation: (**a**,**b**) graphite surface covered by a dense layer of solidified electrolyte; (**c**) a large industrial coke particle anchored via arc-induced nucleation; (**d**) fine carbon particles forming a porous scaffold.

The microscopic investigation captured the initiation and growth of the deposits, revealing a transition from individual particle capture to the formation of a stable composite:

- Initial adhesion and AE-polishing (Figure 9a,b): The early stage is characterized by the “anode effect polishing,” where the graphite surface becomes microscopically smoothed. This smoothing, caused by the intense micro-arc discharges, creates a prepared substrate for the arrival of carbon dust. Driven by horizontal electrophoretic forces, negatively charged coke particles migrate from the bulk electrolyte to this vertical face.
- Arc-induced nucleation (Welding) (Figure 9c): As shown in the high-magnification image of an individual industrial coke particle, the attachment is not merely mechanical. The grain is firmly “welded” into the graphite substrate. This arc-induced nucleation provides the necessary thermal energy to fuse the exogenous carbon grain to the anode, forming the primary conductive nucleus (the spike core).
- Scaffold formation and electrolyte infiltration (Figure 9d): At higher magnifications, a complex, “sponge-like” porous scaffold is visible. This structure is built from the cumulative aggradation of fine carbon particles. Crucially, the “solidified electrolyte” observed in this matrix is the result of capillary infiltration. The liquid acidic melt (chiolite-rich) penetrates the interstitial pores of the carbon scaffold during the AE.

### 4.4. Chemical Confirmation and Mapping

**Figure 10:**
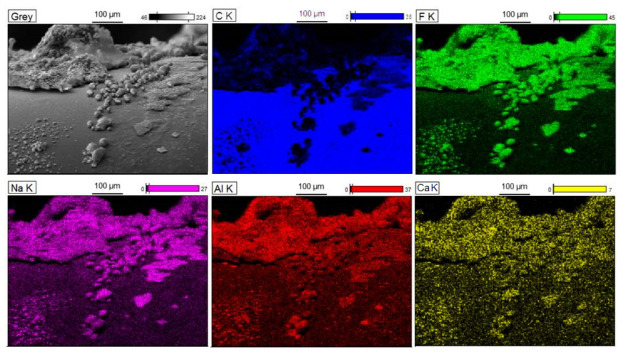
EDS elemental mapping analysis of the anode surface (Exp. No. 1). The spatial correlation between C, F, Na, and Al confirms the formation of a carbon–electrolyte composite.

Elemental mapping via EDS (Figure 10) provided detailed chemical confirmation of this structure. The maps show a perfect spatial correlation between the carbon (C) clusters and the primary bath constituents: Fluorine (F), Sodium (Na), and Aluminum (Al). Notably, certain carbon particles are partially or fully masked by the overlying fluoride phases, proving that the acidic electrolyte infiltrated the porous carbon scaffold while in a liquid state.

### 4.5. Trace Marker Identification

**Figure 11:**
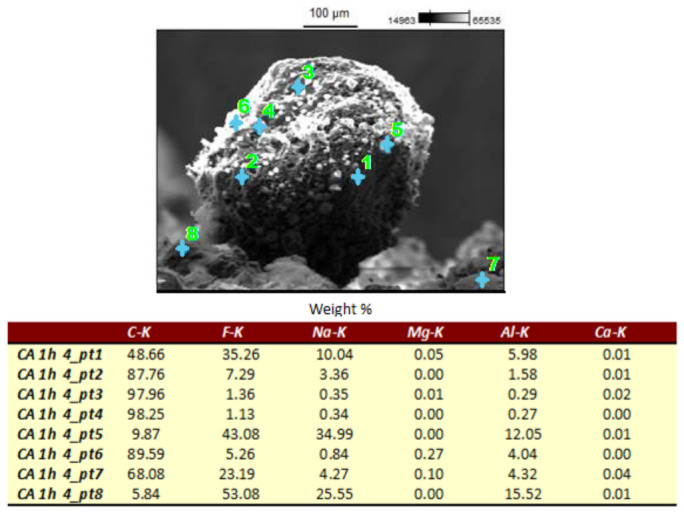
High-magnification SEM image and energy-dispersive X-ray spectroscopy (EDS) point analysis (pt1–pt8) of a redeposited carbon dust particle after a 60 min AE test. All elemental concentration values displayed in the accompanying table are expressed in weight percentages (wt.%).

The most critical evidence of the deposits’ origin comes from the EDS point analysis (Figure 11). Detailed measurements at points pt1–pt8 revealed the systematic presence of Magnesium (Mg) and Calcium (Ca) within the carbon clusters:

Origin Proof: Since the high-purity graphite substrate is free of these elements, their detection (e.g., 0.27 wt.% Mg in pt6) serves as an unambiguous “chemical signature” of industrial dust. This proves that the deposits are redeposited industrial carbon dust (skimmings) rather than products of anode erosion.

Infiltration Evidence: High concentrations of fluoride species in deep interstitial points (e.g., over 50 wt.% F in Figure 11, pt8) confirm that the molten electrolyte was drawn into the carbon matrix via capillary action during the AE.

### 4.6. Kinetics of Growth (60 Min vs. 120 Min)

**Figure 12:**
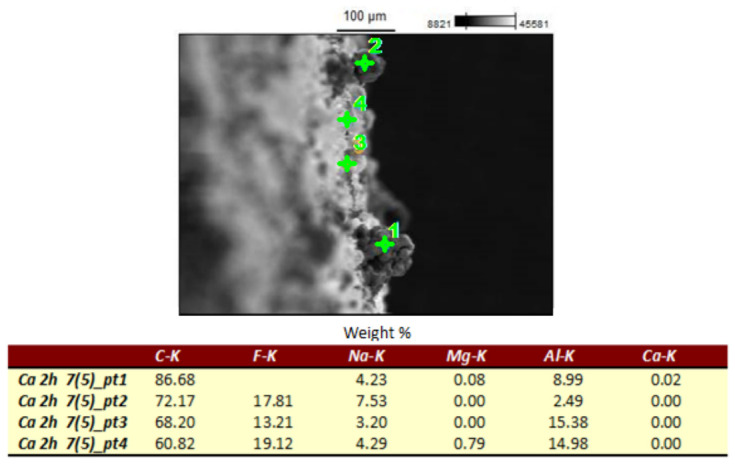
SEM micrograph and energy-dispersive X-ray spectroscopy (EDS) point analysis (pt1–pt4) after a 120 min AE test. All elemental concentration values displayed in the accompanying table are expressed in weight percentages (wt.%).

Comparing the 60 min (Exp. 1) and 120 min (Exp. 2)trials reveals the cumulative nature of the process. In the 120 min samples (Figure 12), the carbonaceous agglomerates are significantly more massive and exhibit a higher degree of compaction. The Mg marker concentration increases (up to 0.79 wt.% Mg), documenting a continuous, field-driven influx of exogenous material. This documents that the longer the anode effect remains unsuppressed, the more the initial carbon nucleus transforms into a stable, dense, and conductive carbon–matrix composite spike.

### 4.7. XRD Structural Characterization

**Figure 13:**
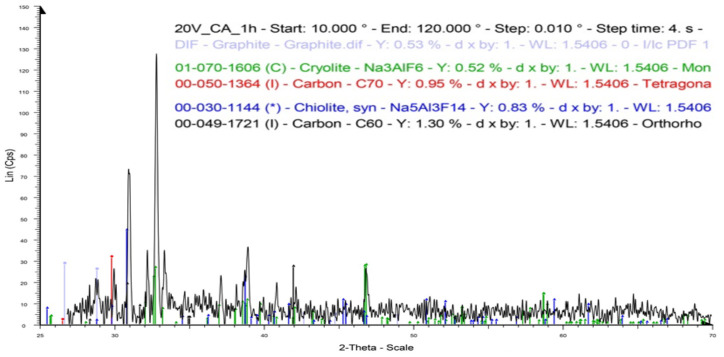
XRD diffractogram of the raw industrial carbon dust (skimmings) used as a tracer, showing characteristic broad reflections of disordered carbonaceous phases (C60/C70 varieties).

**Figure 14:**
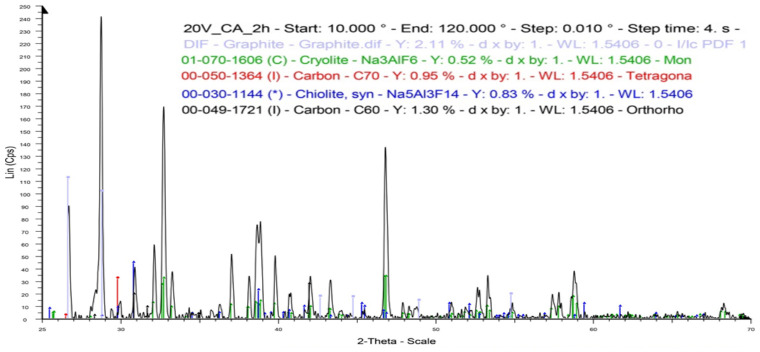
XRD diffractogram of the material collected from the anode surface after a 60 min AE test. The analysis illustrates the multi-phase coexistence of the crystalline graphite substrate, cryolite (Na_3_AlF_6_, green markers), and chiolite (Na_5_Al_3_F_14_, blue markers).

**Figure 15:**
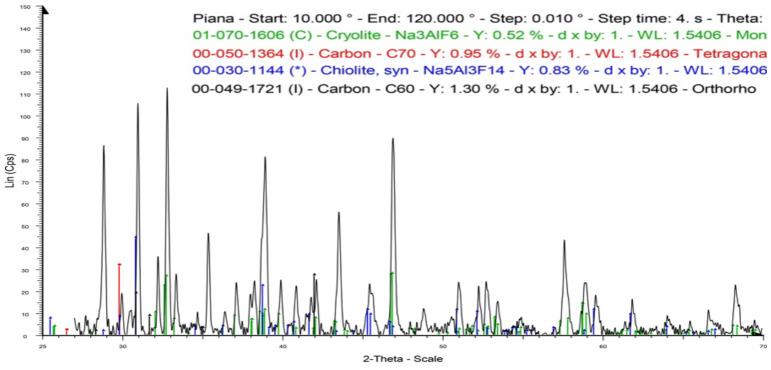
XRD diffractogram of the material collected from the anode surface after a 120 min AE test. The analysis illustrates the intensified presence of cryolite (Na_3_AlF_6_), green markers) and chiolite (Na_5_Al_3_F_14,_ blue markers) phases within the crystalline graphite substrate.

X-ray diffraction (XRD) analysis of the material collected from the anode surface provided definitive structural confirmation of the multi-phase nature of the deposits. To establish a robust analytical baseline, the raw industrial carbon dust (skimmings) was first characterized (Figure 13), followed by the investigation of the material scraped from the vertical anode surface after 60 min (Figure 14) and 120 min (Figure 15) AE tests.

The diffractogram of the raw industrial dust (Figure 13) reveals a predominantly disordered carbonaceous structure, characterized by broad reflections corresponding to amorphous coke phases (identified in the diffraction databases as C60/C70 varieties). As documented by Vander Wal et al. [21], the absence of an intense, sharp (002) reflection and the emergence of this diffuse diffraction halo are structural prerequisites of highly amorphized carbon structures. These broad features correspond to amorphous coke varieties, serving as a definitive structural fingerprint of the industrial carbon dust [21]. The consistency of these disordered carbon signatures between the raw skimmings and the subsequent anode deposits provides an independent structural proof—complementary to EDS analysis—that the spikes are formed by the redeposition of exogenous dust rather than the structural degradation or recrystallization of the ordered graphite substrate.

Upon analysis of the material collected after the 60 min AE test (Figure 14), a complex multi-phase composition was identified. While the sharp, dominant peaks at 2θ angles of approximately 26.5°, 44.4°, and 54.5° originate from the highly crystalline lattice of the isostatic graphite matrix (ICDD PDF No. 00-056-0159), additional reflections confirm the coexistence of cryolite (Na_3_AlF_6_) and chiolite (Na_5_Al_3_F_14_). Cryolite was successfully indexed via characteristic peaks at 2θ values of 19.3°, 22.4°, 31.2°, and 32.4° (ICDD PDF No. 01-073-1387), whereas chiolite was definitively confirmed by distinct reflections at 2θ positions of 16.2°, 27.5°, 29.1°, and 31.9° (ICDD PDF No. 01-070-3498). As established by Thonstad et al., the presence of chiolite is a direct structural indicator of the localized acidification of the anode diffusion layer. This acidification occurs due to the rapid depletion of alumina and the subsequent increase in AlF_3_ concentration during the anode effect.

The comparative analysis of the 120 min sample (Figure 15) shows a marked evolution in the intensity of the fluoride phases. The significant increase in the relative intensity of the chiolite reflections (marked in blue) is quantitatively confirmed by the change in the relative peak intensity ratio (I_Chiolite_/I_Graphite_), calculated using the primary chiolite reflection at 2θ ≈ 16.2° and the dominant graphite peak at 2θ ≈ 26.5°. This ratio increased from approximately 0.06 in the 60 min trial to 0.13 in the 120 min sample, documenting a cumulative infiltration process. During the extended AE, the sustained shift in bath chemistry toward the chiolite stability field (CR < 2.2) facilitates a deeper saturation of the porous carbon scaffold.

These results strongly support the capillary infiltration model. Due to its lower melting point (~730 °C) compared to pure cryolite (~1010 °C), the chiolite-rich acidic melt remains in a highly fluid state during the arc-induced thermal anomalies of the AE. This allows the electrolyte to penetrate the interstitial voids of the “welded” carbon nucleus. The chiolite identified in the sampled material thus represents the solidified product of the infiltrated melt, which provides the final mechanical robustness to and density of the initiated carbon–electrolyte composite spike.

### 4.8. Raman Spectroscopy: Evaluation of Carbon Allotropes

**Figure 16:**
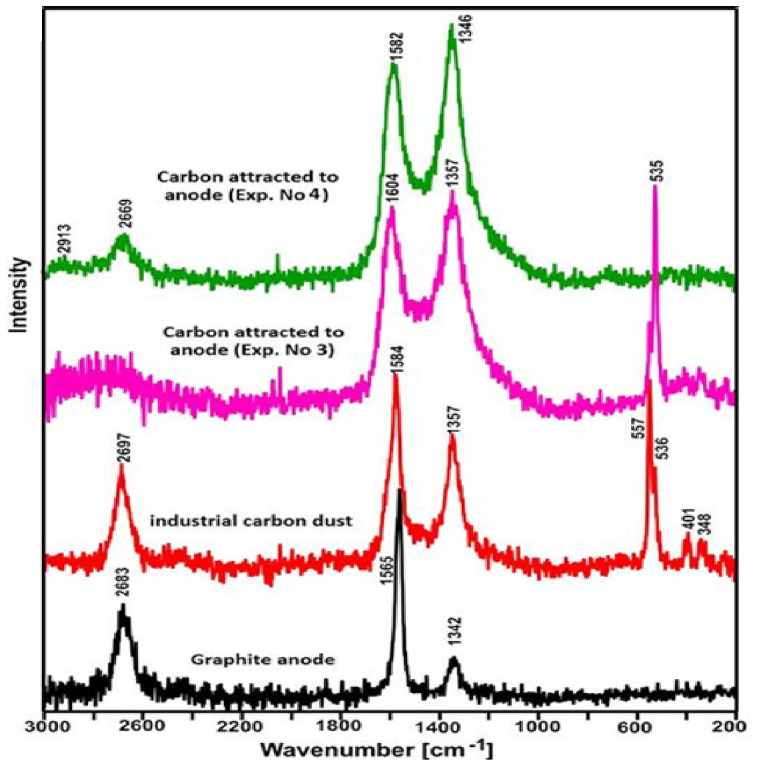
Comparative Raman spectra of the isostatic graphite substrate (baseline), the raw industrial carbon dust (skimmings), and the deposits captured on the anode surface during Experiment No. 1 and No. 2. Characteristic carbon bands (D, G, 2D) and low-frequency fluoride modes (200–600 cm^−1^) are marked with their respective Raman shifts.

Raman spectroscopy was employed to evaluate the structural ordering of the carbonaceous matter and to distinguish between the primary graphite substrate and the redeposited industrial dust. The comparative analysis of the Raman spectra (Figure 16) provides a “structural fingerprint” that confirms the exogenous origin of the initiated spikes.

The Raman spectrum of the Graphite anode (baseline) is dominated by a sharp, intense G-band at 1582 cm^−1^, which corresponds to the ideal sp^2^ hexagonal lattice. The secondary 2D-band (G’) at 2683 cm^−1^ is well-defined, further confirming the high degree of crystalline order of the isostatic graphite. The low intensity of the defect-induced D-band (~1342 cm^−1^) in the substrate serves as a clear reference point, representing the pristine state of the electrode.

In radical contrast, the spectrum of the industrial carbon dust (tracer) and the deposits captured in Experiment No. 1 and No. 2 show a profound structural transformation. The most prominent feature of these deposits is the emergence of a very intense and broad D-band at approximately 1346–1357 cm^−1^. According to the structural criteria established by Sadezky et al. [12], this high intensity of the D-band relative to the G-band is quantitatively confirmed by the I_D_/I_G_ ratio. While the pristine graphite anode exhibits an I_D_/I_G_ ratio below 0.05, the ratios for the raw industrial tracer and the redeposited anode growth surge to 0.96 and 1.05, respectively. This high intensity ratio (I_D_/I_G_) is a definitive indicator of highly disordered carbon structures, characteristic of industrial coke that has undergone fragmentation and selective oxidation.

### 4.9. Key Observations from the Comparative Analysis Include

- Structural heredity: The spectra of the carbon attracted to the anode are nearly identical to the signature of the industrial carbon dust tracer. Both exhibit the broad D and G bands typical of amorphous carbon, proving that the spikes are built from the redeposition of exogenous skimmings rather than the recrystallization of the electrode material.
- Presence of fluoride phases: The anode deposits and the industrial dust show distinct low-frequency peaks in the 200–600 cm^−1^ range. As documented by Ishak et al. [13], these features are attributed to integrated fluoride species (cryolite and chiolite) within the carbon matrix. The coexistence of these mineral signals with the disordered carbon peaks provides microscopic evidence of the composite nature of the initiated spike.

These results unequivocally demonstrate that the anode effect triggers the rapid aggradation of disordered industrial dust, which serves as the conductive nucleus for spike growth. The Raman investigation perfectly correlates with the broad C60/C70 reflections observed in the XRD analysis, providing a multiscale confirmation of the spike initiation mechanism. Ultimately, this multiscale correlation establishes a clear analytical hierarchy: the profound contrast between the highly disordered carbon lattice of the deposits (I_D_/I_G_ ≈ 1.0) and the pristine sp^2^ matrix of the substrate I_D_/I_G_ < 0.05) serves as the primary structural proof of the growth’s exogenous origin. The subsequent tracking of sub-percent Mg and Ca chemical markers—which perfectly match the low elemental baselines dictated by industrial bath chemistry—acts as a secondary chemical seal, confirming that the deposited amorphous coke blocks are authentic industrial skimmings attracted from the melt via electrophoretic transport [12,21].

### 4.10. Confocal Microscopy

Laser confocal microscopy was employed to investigate the topographical evolution and the spatial relationship between the redeposited carbon dust and the electrolyte phase. By comparing the raw industrial tracer with the deposits captured after different AE durations, a clear “structural heredity” was established.

### 4.11. Baseline Characterization of Industrial Dust

**Figure 17:**
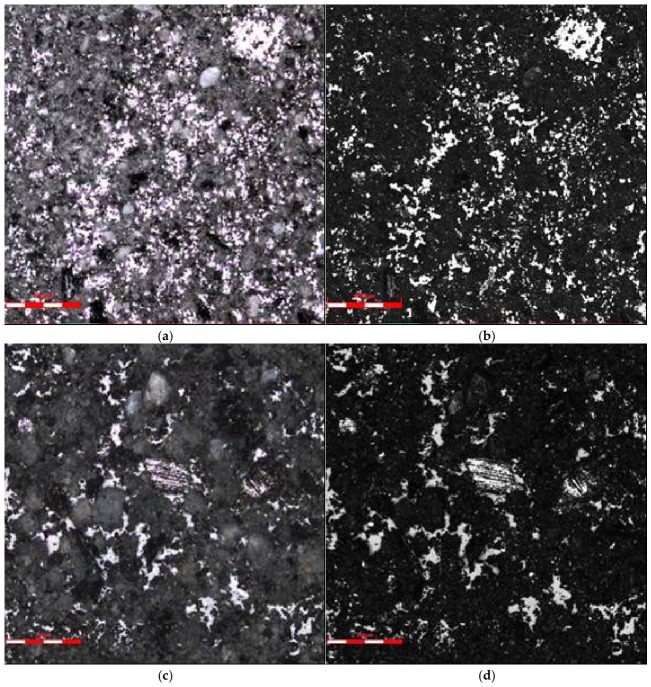
Confocal microscopy images of raw industrial carbon dust (skimmings) from an operational smelter: (**a**,**c**) polarized light; (**b**,**d**) laser light. The scale bar represents 50 µm.

To establish a definitive structural baseline, the raw industrial carbon dust (skimmings) collected directly from the smelter was first analyzed (Figure 17). The micrographs in polarized light (a,c) and laser light (b,d) reveal a highly heterogeneous and porous architecture. The bright, reflective spots within the dark coke grains indicate that the industrial dust is already pre-impregnated with fluoride phases from its residence in the smelter bath. As suggested by Bugnion & Fischer [10], this pre-existing electrolyte within the carbon pores significantly influences the dust’s electrophoretic behavior and its ability to nucleate on the electrode surface.

### 4.12. Early Stage: 60-Minute AE Exposure

**Figure 18:**
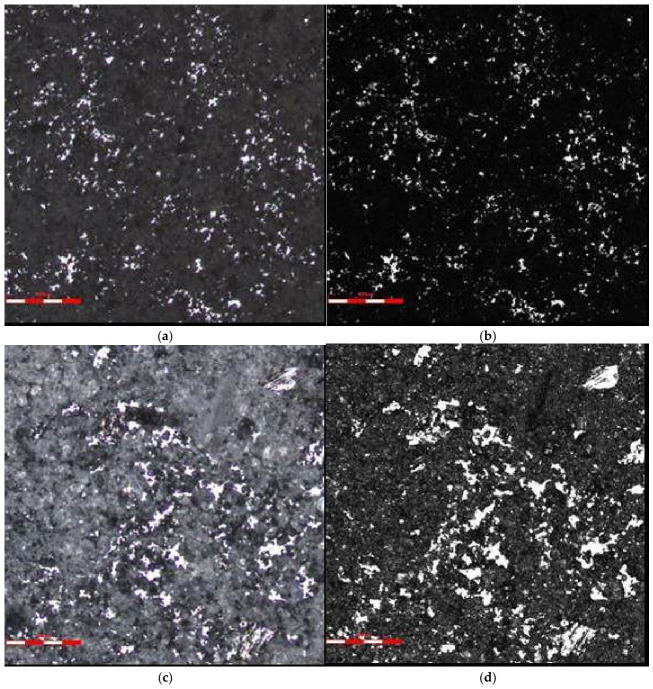
Laser confocal microscopy images of the anode surface after a 60 min AE test: (**a**,**c**) polarized light; (**b**,**d**) laser light. The scale bar represents 20 µm.

The images for the 60 min AE test (Figure 18) provide a high-contrast visualization of the initial stages of spike formation. The surface is characterized by isolated, bright crystalline clusters (solidified fluoride phases) dispersed within the darker carbonaceous matrix. At this stage, the carbonaceous particles do not form a continuous film but act as individual “islands” of nucleation. The laser light images (Figure 18b,d) highlight the porous architecture of the deposits, providing further evidence for the capillary infiltration model, where the molten acidic electrolyte was drawn into the interstitial spaces of the arc-welded carbon particles before solidifying.

### 4.13. Advanced Stage: 120-Minute AE Exposure

**Figure 19:**
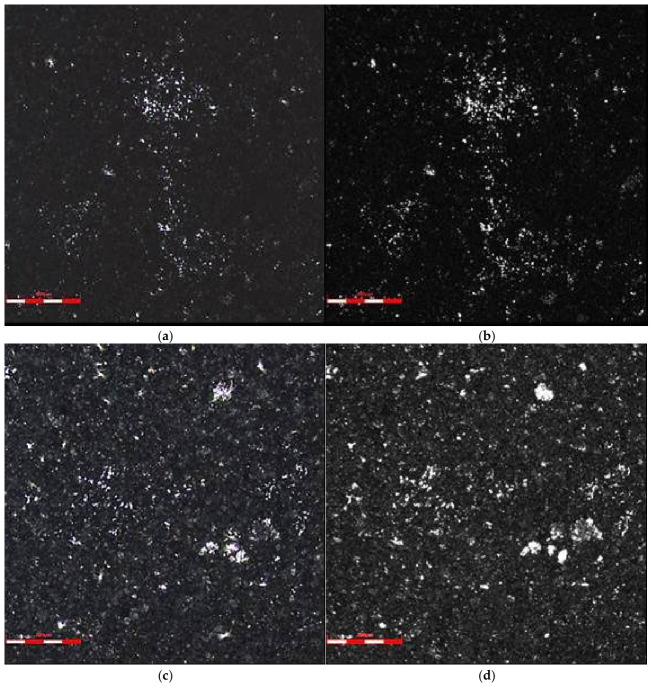
Laser confocal microscopy images of the anode surface after a 120 min AE test: (**a**,**c**) polarized light; (**b**,**d**) laser light. The scale bar represents 20 µm.

The analysis after 120 min of AE exposure (Figure 19) reveals a significant progression in topographical complexity. The micrographs document a transition from isolated clusters toward massive aggradation and interconnected growth. As noted by Ishak et al. [13], the increase in AE duration facilitates the continuous capture of additional carbon dust, leading to the topographical “bridging” of previously isolated nuclei. The laser light images (Figure 19b,d) show that the fluoride reflections have formed a continuous skeletal network that reinforces the carbon scaffold, representing a stable, conductive, and mechanically robust industrial spike.

### 4.14. Effect of AE Duration on Spike Growth

The comparative analysis of samples from the 60 and 120 min experiments demonstrates that the duration of the anode effect is a decisive factor in the initiation and stabilization of anode spikes. Multiscale investigations revealed a clear progression in both the mass and the structural complexity of the deposits as a function of time.

While the 60 min exposure resulted in the formation of isolated, relatively thin carbon clusters, the 120 min test led to the development of massive, three-dimensional agglomerates that significantly protruded from the anode plane. As described by Bugnion and Fischer [7] in their “Vicious Circle” theory, this growth is a self-reinforcing process: once the initial carbon nucleus is “welded” to the anode, it locally reduces the interpolar distance, which further intensifies the electric field and micro-arc discharges, accelerating the capture of additional dust particles.

XRD data further confirmed this temporal progression, showing a marked increase in the relative intensity of chiolite and cryolite reflections for the longer duration. This indicates a more advanced stage of capillary infiltration, where the acidic melt progressively saturates the porous carbon scaffold. According to Thonstad et al. [2], the prolonged duration of the anode effect leads to an increased accumulation of AlF_3_-rich species in the diffusion layer, which explains the higher concentration of solidified fluoride phases (chiolite) observed in our 120 min samples.

These results confirm the cumulative nature of the electrophoretic transport process. The continuous influence of the intense electric field and the localized thermal anomalies from micro-arcs facilitate the successive layering of industrial dust. Consequently, the longer an anode effect remains unsuppressed, the higher the probability of transforming a localized dust cluster into a stable, conductive carbon–matrix composite spike capable of causing metallic short circuits in an industrial cell.

## 5. General Discussion

### 5.1. Validation of the Electrophoretic Transport Hypothesis

The primary objective of this study was to isolate and confirm the electrokinetic transport of carbon dust in a cryolite melt. The implementation of a unique vertical electrode setup successfully eliminated the “sedimentation bias” common in horizontal cell designs. The macroscopic observations (Figure 7) provided the first physical proof: the accumulation of carbonaceous matter occurred exclusively on the vertical anode face, defying gravitational settling.

The detection of magnesium (Mg) and calcium (Ca) markers via EDS point analysis (Figure 11 and Figure 12) serves as an unambiguous “chemical signature” of the redeposited material. Since these markers are intrinsic to the industrial skimmings (Figure 17) and absent in the high-purity graphite substrate (Figure 8a), their presence on the anode surface confirms that the spikes are built from exogenous dust particles transported across the 4.5 cm interpolar gap by intense electrophoretic forces during the anode effect (AE). This horizontal migration under high electric field intensity aligns with the electrokinetic principles established by Polyakov et al. [9].

### 5.2. Mechanism of Spike Initiation: Arc-Induced Nucleation and Composite Growth

Based on the integrated multiscale analysis, a two-stage mechanism for spike initiation is proposed:

- Stage 1: Arc-induced nucleation and “welding”

The initiation is triggered by micro-arc discharges that pierce the insulating gas film during the AE (Figure 5 and Figure 6). These high-energy events force industrial carbon particles into the graphite irregularities, creating a direct carbon-to-carbon “welded” scaffold. The Raman spectra (Figure 16) and XRD disordered phases (Figure 14) confirm that this scaffold is composed of amorphous industrial coke, distinctly different from the ordered graphite electrode. The structural disorder of the redeposited scaffold, confirmed by the wide diffraction bands typical of soot-like carbons [21], proves the exogenous origin of the spike core. The early-stage adhesion of individual particles on the “AE-polished” surface (Figure 8b and Figure 9c) provides a clear visualization of these primary nucleation sites. This micro-smoothing eliminates surface asperities, establishing a geometrically uniform gas film that stabilizes the dielectric breakdown. Consequently, the flattened topography maximizes the true contact area between the arriving exogenous particles and the anode substrate, lowering interface resistance. As suggested by Gao et al. [11], the localized plasma formation during these discharges provides the thermal density required for such structural fusion. Furthermore, the surface composition of the substrate plays a defining role in these fusion kinetics. While the high-purity isostatic graphite used here establishes a conservative, chemically inert carbon baseline, authentic industrial prebaked anodes present a highly heterogeneous surface rich in heteroatoms (S, O, N) and metallic impurities (V, Ni, Fe). These chemical impurities act as active chemisorption sites that lower the local energy threshold for arc-induced nucleation, implying that the raw surface composition of smelter anodes would further accelerate the chemical anchoring of the captured industrial dust.

- Stage 2: Capillary infiltration and structural stabilization

Based on these post-mortem findings, a conceptual model for the high-temperature stability of the spike can be proposed. While the solid, arc-welded carbon skeleton provides the primary mechanical resistance against the bulk electrolyte flow, the subsequent capillary infiltration of the acidic melt acts as a hydrodynamic shield at 975 °C. Fluid confined within the micro-pores is protected from external convection by boundary-layer viscous dissipation. This liquid-filled matrix prevents the turbulent bath from directly tearing apart the internal carbon–carbon welds, holding the structural integrity of the growth until post-test quenching crystallizes the trapped liquid into the diagnostic chiolite phase (Thonstad et al. [2]; Robelin & Chartrand [17]).

### 5.3. Progression of Growth and Industrial Implications

The comparative analysis of 60 and 120 min trials documents a cumulative and non-linear growth process. Confocal microscopy (Figure 19) visualized the critical transition from isolated, unstable clusters to interconnected, three-dimensional “islands” that form a continuous conductive path. The systematic increase in Mg marker concentration in longer trials (Figure 12), coupled with the intensification of chiolite reflections in XRD diffractograms (Figure 15), proves that the longer an anode effect (AE) remains unsuppressed, the more robust and dense the carbon–electrolyte composite becomes.

This process follows the “Vicious Circle” kinetics described by Bugnion and Fischer [7], where the initial “welded” carbon nucleus reduces the local interpolar distance, leading to localized current concentration and further thermal anomalies. From an industrial perspective, these findings emphasize that the “spike” is not a static object but a dynamic, self-reinforcing structure. The rapid infiltration of the acidic melt (chiolite) provides the final mechanical anchoring, making the spike resistant to the turbulent flow of the electrolyte. Therefore, the critical necessity for rapid AE termination—ideally within seconds—is not only a matter of energy efficiency but a fundamental requirement to prevent the physical stabilization of these conductive growths, which are the primary cause of downstream current maldistribution and metallic short circuits. While laboratory scales require extended durations due to the absence of high-amperage electromagnetic convection, the underlying electrokinetic anchoring and micro-arc welding occur almost instantaneously, validating the application of this mechanism to industrial timescales. Furthermore, it must be noted that while high-purity isostatic graphite was required for analytical baseline clarity, the structural heterogeneity and inherent porosity (20–25%) of authentic industrial prebaked anodes are expected to heavily accelerate the spike initiation cascade. The complex aggregate–binder interfaces in industrial anodes generate localized electrical hotspots that lower the macro-voltage threshold required for arc-induced welding. Simultaneously, the open surface porosity acts as a geometric trap for electrophoretically captured dust while enhancing the depth of capillary infiltration by the acidic chiolite-rich melt, resulting in a more deeply anchored and mechanically robust composite growth under smelter conditions.

From a diagnostic perspective, the transition into the spike initiation regime is governed by a critical force balance where the horizontal electrophoretic vector overrides the turbulent hydrodynamic drag of the electrolyte flow. Under normal cell operation (~1.0 V/cm field intensity), turbulence easily shears away migrating particles. However, when local alumina depletion drives voltage anomalies, exceeding a critical localized electric field threshold (estimated at >3.5 V/cm), electrophoretic forces become dominant. This shift marks the precise operational flashpoint where suspended dust can overcome boundary-layer turbulence and achieve the structural contact required for arc-induced welding.

### 5.4. Future Research Directions

While this study establishes the fundamental mechanism of arc-induced nucleation and electrophoretic transport, further research is needed to broaden the industrial application of these findings. Future investigations should focus on:

- Physicochemical mitigation: Evaluating the impact of different electrolyte additives on the surface charge of carbon particles to minimize their electrophoretic mobility.
- Material optimization: Analyzing how industrial anode formulations and baking parameters influence the resistance to arc-induced “welding.”
- Diagnostic tools: Exploring the potential for real-time monitoring of micro-arc discharge frequencies as a predictive indicator of early-stage spike initiation.

## 6. Conclusions

The experimental investigation conducted using a unique vertical electrode configuration and multiscale structural analysis led to the following conclusions regarding the mechanism of anode spike initiation:

- Validation of electrophoretic transport: The study provides unambiguous physical proof that the initiation of anode spikes is driven by electrokinetic forces. By eliminating gravitational sedimentation through a vertical electrode arrangement, it was demonstrated that carbon dust particles undergo horizontal electrophoretic migration across the interpolar gap, anchored exclusively to the vertical anode faces.
- Exogenous origin of deposits: The use of industrial skimmings as a tracer, combined with the identification of Mg and Ca chemical markers on high-purity graphite substrates, confirms that the spikes are formed by the capture of exogenous industrial dust rather than the degradation or recrystallization of the anode material.
- Arc-induced nucleation (Welding): The transition from loose suspended particles to a permanent deposit is governed by arc-induced nucleation. Micro-arc discharges during the anode effect provide the necessary thermal energy to “weld” industrial coke particles into a conductive scaffold, creating the primary nucleus of the spike.
- Composite nature of spikes: Structural characterization via XRD, Raman, and confocal microscopy confirms that anode spikes are carbon–electrolyte composites. The porous carbon scaffold, built from disordered industrial coke, is subsequently infiltrated by an acidic fluoride melt. The presence of chiolite (Na_5_Al_3_F_14_) serves as a structural indicator of localized acidification and capillary infiltration during the growth process.
- Cumulative growth kinetics: The duration of the anode effect is a decisive factor in spike stabilization. Comparative analysis (60 vs. 120 min) shows a progression from isolated clusters to massive, interconnected three-dimensional agglomerates. This self-reinforcing “vicious circle” process highlights the critical importance of rapid AE suppression to prevent the formation of stable, conductive growths.
- Structural heredity: Raman spectroscopy proved a clear structural heredity between the industrial tracer and the anode deposits. The high degree of structural disorder (I_D_/I_G_ ratio) and the presence of integrated fluoride phases distinguish these secondary growths from the ordered graphite matrix.

## Data Availability

The original contributions presented in this study are included in the article. Further inquiries can be directed to the corresponding author.
